# Factors associated with kinesiophobia in patients with inflammatory bowel disease: associations with functional independence and fatigue

**DOI:** 10.3389/fmed.2026.1900689

**Published:** 2026-08-14

**Authors:** Xinyi Gao, Yi Wang, Xinxin Shao, Yunzheng Di, Rong Wang, Yamei Chen

**Affiliations:** 1 School of Nursing, Anhui University of Chinese Medicine, Hefei, China; 2 Department of Nursing, Shanghai Tenth People's Hospital, Tongji University School of Medicine, Shanghai, China

**Keywords:** fatigue, functional independence, inflammatory bowel disease, kinesiophobia, physical activity

## Abstract

**Background:**

Patients with inflammatory bowel disease (IBD) often have low levels of physical activity, which may be related to kinesiophobia. However, the associations of independence in basic activities of daily living, fatigue, and disease activity with kinesiophobia remain insufficiently understood. This study examined factors associated with kinesiophobia in patients with IBD, focusing on independence in basic activities of daily living and fatigue.

**Methods:**

A cross-sectional study included 249 patients with IBD recruited from a tertiary hospital in Shanghai, China, between June and October 2025. Kinesiophobia was assessed using the Tampa Scale for Kinesiophobia-17 (TSK-17), independence in basic activities of daily living using the Barthel Index, and fatigue using the Multidimensional Fatigue Inventory (MFI-20). Disease activity was assessed using disease-specific clinical indices and classified into common activity categories. Multiple linear regression analyses were used to examine factors associated with kinesiophobia. An expanded covariate-adjusted model and sensitivity analyses assessed consistency of findings.

**Results:**

The mean TSK-17 score was 38.84 ± 10.02, and 142 patients (57.0%) scored at or above the descriptive threshold of 37. Lower independence in basic activities of daily living (B = −0.284, β = −0.561, *P* < 0.001) and greater fatigue (B = 0.097, β = 0.171, *P* = 0.001) were associated with greater kinesiophobia. The Barthel Index had the largest absolute standardized coefficient. Female sex and medical insurance type were associated with kinesiophobia. Moderate disease activity was associated with greater kinesiophobia than lower clinical activity (remission or mild disease activity), whereas severe disease activity was not. The associations with independence in basic activities of daily living and fatigue remained significant across expanded and sensitivity analyses, whereas the association with disease activity varied across analytical approaches.

**Conclusion:**

Kinesiophobia in patients with IBD was more consistently associated with lower independence in basic activities of daily living and greater fatigue than with disease activity. These findings underscore the relevance of patients' everyday functioning and symptom burden when evaluating movement-related concerns. Considering these dimensions alongside disease activity may support more comprehensive assessment of movement-related concerns and a more individualized approach to physical activity in IBD care.

## 1 Introduction

Inflammatory bowel disease (IBD), primarily including Crohn's disease (CD) and ulcerative colitis (UC), is a chronic, relapsing inflammatory disorder of the gastrointestinal tract characterized by recurrent disease activity and impaired physical and psychosocial functioning (1). Although the exact etiology of IBD remains incompletely understood, current evidence suggests that genetic susceptibility, environmental factors, gut microbiota dysbiosis, and immune dysfunction jointly contribute to disease development (2). Over the past decades, the incidence of IBD has increased substantially worldwide, particularly in newly industrialized countries. In China, the age-standardized incidence of IBD increased from 1.47 per 100,000 in 1990 to 3.01 per 100,000 in 2019, representing an increase of more than 100% (1, 3). Clinically, IBD is associated not only with gastrointestinal symptoms such as abdominal pain, diarrhea, and bloody stools, but also with multiple systemic manifestations and extraintestinal complications, including musculoskeletal symptoms, malnutrition, anemia, fatigue, anxiety, and depression (4). Even during clinical remission, many patients continue to experience persistent symptoms and impaired daily functioning, resulting in substantially reduced quality of life (5).

The primary goals of IBD management are to control disease activity, maintain long-term remission, reduce complications, and improve patients' physical and psychosocial wellbeing (6). Although pharmacological therapies remain the cornerstone of treatment, many patients continue to experience recurrent relapses, persistent fatigue, and impaired daily functioning despite advances in biologic and targeted therapies (7). Consequently, increasing attention has been directed toward non-pharmacological interventions as complementary strategies for long-term disease management (8). Among these, physical activity has emerged as a promising adjunctive approach because of its potential physical and psychological benefits (9). Previous studies have demonstrated that regular exercise may reduce fatigue, improve physical function and muscle strength, alleviate anxiety and depressive symptoms, and enhance health-related quality of life in patients with IBD (10). In addition, structured exercise programs, when appropriately prescribed, have been shown to be safe and not to exacerbate disease activity in patients with clinically stable or mildly active IBD, supporting their role as a feasible adjunctive approach (9, 11).

Despite the recognized benefits of exercise, many patients with IBD continue to exhibit low levels of physical activity (9, 12). Previous studies have shown that patients frequently reduce exercise participation after diagnosis, and most tend to engage only in low-intensity activities such as walking (13). This phenomenon may be associated with kinesiophobia, which is defined as an excessive and irrational fear of movement or physical activity resulting from concerns about pain, symptom exacerbation, or disease relapse (12). In patients with chronic diseases, kinesiophobia has been associated with reduced physical activity, functional decline, poorer psychological well-being, and lower quality of life (14–16). For patients with IBD, recurrent abdominal symptoms, fatigue, bowel urgency, and uncertainty regarding disease progression may occur alongside movement-related fear and activity avoidance (17, 18). Even during remission, some patients remain concerned that exercise may aggravate symptoms or trigger disease recurrence, which may limit their physical activity participation (18). Kinesiophobia, reduced physical activity, physical deconditioning, and fatigue may therefore represent closely interrelated experiences in this population (19).

Functional independence and fatigue may be important factors associated with kinesiophobia in patients with IBD (12, 20). Functional independence refers to an individual's ability to perform basic activities of daily living without assistance (21). Lower independence in basic activities of daily living may involve limitations in routine mobility and self-care. Such limitations may be accompanied by lower perceived physical capability and greater movement-related concerns. Fatigue is one of the most common and burdensome symptoms experienced by patients with IBD and may persist even during periods of clinical remission (22). Persistent fatigue may be associated with lower perceived capacity for physical activity and greater concerns regarding physical exertion or symptom aggravation (18). Therefore, lower functional independence and greater fatigue may both be relevant to movement-related fear in patients with IBD.

Recent studies have begun to investigate kinesiophobia in patients with IBD and have identified fatigue as an important factor associated with fear of movement (12). However, existing research has primarily examined the levels and clinical correlates of kinesiophobia, while the association between functional independence and movement-related fear remains insufficiently understood. In particular, the associations of functional independence, fatigue, and clinical disease activity with kinesiophobia have not been adequately clarified. Therefore, this study aimed to examine factors associated with kinesiophobia in patients with IBD, with particular attention to functional independence and fatigue. A clearer understanding of these associations may support a more comprehensive assessment of movement-related concerns in patients with IBD.

## 2 Methods

### 2.1 Study design and setting

A cross-sectional study was conducted at Shanghai Tenth People's Hospital, Tongji University, Shanghai, China, from June to October 2025. Patients with IBD were recruited from the gastroenterology inpatient ward of this tertiary hospital, which serves more than 1,500 patients with IBD annually. Potentially eligible patients were screened by two trained research nurses. After eligibility assessment, the study purpose and procedures were explained in detail to eligible patients, and written informed consent was obtained before participation. Participants independently completed a paper-based questionnaire booklet, with an average completion time of approximately 20 min. The questionnaires were checked immediately after completion by the research nurses for completeness. The Strengthening the Reporting of Observational Studies in Epidemiology (STROBE) checklist was followed to guide the reporting of the study (23). Ethical approval was obtained from the Medical Ethics Committee of Shanghai Tenth People's Hospital (approval No. 22K233).

### 2.2 Participants

Participants were included based on the following criteria: (1) a confirmed diagnosis of inflammatory bowel disease (Crohn's disease or ulcerative colitis); (2) age ≥18 years; and (3) voluntary participation and provision of written informed consent. Participants were excluded based on: (1) impaired consciousness or hearing impairment; (2) a documented history of psychiatric disorders or a family history of severe mental illness; (3) severe comorbid conditions involving major organs (heart, lungs, brain, or others); (4) unstable condition following surgery; or (5) conditions known to significantly affect physical activity, including cardiovascular and cerebrovascular diseases, chronic obstructive pulmonary disease, malignant tumors, or lower extremity venous thrombosis.

### 2.3 Sample size

*A priori* power analysis was performed using G^*^Power version 3.1.9.7 to estimate the minimum required sample size for multiple linear regression analysis. Assuming a medium effect size (f^2^ = 0.15), a significance level of 0.05, a statistical power of 0.80, and 11 predictor degrees of freedom in the initially specified model, the minimum required sample size was 123 participants, according to Cohen's guidelines. A total of 249 participants were included in the present study, exceeding the minimum sample size requirement.

### 2.4 Measures

#### 2.4.1 Sociodemographic and clinical variables

Sociodemographic and clinical information was collected using a researcher-designed questionnaire, including age, sex, body mass index (BMI), educational level, marital status, place of residence, living arrangement, employment status, monthly household income per capita, type of medical insurance, disease type, disease duration, cumulative number of IBD-related hospitalizations since diagnosis, medication use, and surgical history.

#### 2.4.2 Disease activity

Disease activity was assessed using disease-specific indices: the Simple Clinical Colitis Activity Index (SCCAI) for ulcerative colitis (24) and the Harvey–Bradshaw Index (HBI) for Crohn's disease (25). Disease activity scores were obtained from the medical records at admission for the index hospitalization. Based on established clinical thresholds, disease activity was categorized as remission, mild, moderate, or severe (SCCAI: 0–2 remission, 3–5 mild, 6–11 moderate, and ≥12 severe; HBI: ≤ 4 remission, 5–7 mild, 8–16 moderate, ≥17 severe). The raw SCCAI and HBI scores were not combined directly. Instead, the disease-specific scores were mapped to a common four-category disease-activity variable for the combined IBD sample.

#### 2.4.3 Barthel index (BI)

Functional independence in basic activities of daily living was assessed using the Barthel Index (BI) (21). The BI was selected because the Chinese national nursing classification standard (WS/T 431–2023) specifies its use for assessing self-care ability among inpatients in general hospitals (26). The BI consists of 10 items assessing feeding, bathing, grooming, dressing, bowel and bladder control, toileting, chair–bed transfer, mobility (ambulation or wheelchair use), and stair climbing. Item scores are 0, 5, 10, or 15 points, depending on the activity, with a total score ranging from 0 to 100. Higher scores indicate greater functional independence. According to this standard, BI scores ≤ 40 indicate severe dependence, scores of 41–60 indicate moderate dependence, scores of 61–99 indicate mild dependence, and a score of 100 indicates complete independence. In this study, the BI was interpreted specifically as a measure of independence in basic activities of daily living. The Cronbach's α coefficient of the BI in the current sample was 0.885.

#### 2.4.4 Tampa scale for kinesiophobia (TSK-17)

Kinesiophobia was assessed using the Tampa Scale for Kinesiophobia (TSK-17), originally developed by Kori et al. to evaluate fear of movement and reinjury beliefs (27). The Chinese version translated and culturally adapted by Hu et al. was used in this study (28). The TSK-17 consists of 17 items rated on a 4-point Likert scale ranging from 1 (“strongly disagree”) to 4 (“strongly agree”). Total scores range from 17 to 68, with higher scores indicating greater fear of movement. A score of ≥37 was used for descriptive classification (29). Because this threshold has not been specifically validated in patients with IBD, it was not interpreted as a diagnostic cutoff. The Chinese version demonstrated a Cronbach's α coefficient of 0.778 and a test–retest reliability of 0.860. In the present study, the TSK-17 demonstrated a Cronbach's α coefficient of 0.938.

#### 2.4.5 Multidimensional fatigue inventory (MFI-20)

Fatigue was measured using the Chinese version of the Multidimensional Fatigue Inventory (MFI-20), originally developed by Smets et al. to assess fatigue experienced over the previous 2 weeks (30). The Chinese version translated and validated by Miao et al. was adopted in this study (31). The MFI-20 comprises 20 items across five dimensions: general fatigue, physical fatigue, mental fatigue, reduced motivation, and reduced activity. Each dimension contains four items. Responses are rated on a 5-point Likert scale ranging from 1 (“completely inconsistent”) to 5 (“completely consistent”). Items 1, 3, 4, 6, 7, 8, 11, 12, 15, and 20 are reverse scored. Total scores range from 20 to 100, with higher scores indicating more severe fatigue. The Chinese version demonstrated a Cronbach's α coefficient of 0.882. In the present study, the Cronbach's α coefficient was 0.969.

### 2.5 Statistical analysis

Statistical analyses were performed using SPSS version 27.0. Normally distributed continuous variables were summarized as means and standard deviations (SDs), whereas non-normally distributed continuous variables were summarized as medians and interquartile ranges (IQRs), and categorical variables as frequencies and percentages. Differences in TSK-17 total scores across categorical variables were examined using independent-samples *t* tests or one-way analysis of variance (ANOVA), as appropriate. Normality was assessed using Shapiro–Wilk tests and inspection of distributional characteristics, and homogeneity of variance was assessed using Levene's test; Welch's *t* test or Welch's ANOVA was used when variances were unequal. Nonparametric sensitivity analyses were performed using the Mann–Whitney U test or Kruskal–Wallis test, as appropriate. Pearson correlation analysis was used to assess the relationships of age and scale total scores with the TSK-17 total score, whereas Spearman rank correlation analysis was used for the cumulative number of IBD-related hospitalizations since diagnosis because of its skewed count distribution. All measured sociodemographic and clinical variables were examined in the univariate analyses.

Multiple linear regression analysis was performed with the TSK-17 total score as the continuous dependent variable. Variables included in the regression model were selected based on the study objectives, clinical relevance, potential confounding effects, and univariate findings; univariate statistical significance was not used as the sole selection criterion. The primary model included sex, age, the cumulative number of IBD-related hospitalizations since diagnosis, disease type, disease activity, type of medical insurance, BI total score, and MFI-20 total score. Age and the cumulative number of hospitalizations were entered as continuous variables. Given the small number of participants in remission (*n* = 11), remission and mild disease activity were combined into a lower clinical activity category in the primary analysis to improve the stability of coefficient estimates and avoid the use of a sparse reference group. This grouping also has precedent in the three-level TELE-IBD monitoring framework, which combined remission and mild disease activity into a lower-activity category while retaining moderate and severe disease activity as separate categories (32, 33). Accordingly, disease activity was entered into the primary model as a three-category variable comprising lower clinical activity (remission or mild disease activity), moderate disease activity, and severe disease activity, with lower clinical activity as the reference category. Categorical variables were dummy-coded, and all variables specified for each model were entered simultaneously.

An expanded covariate-adjusted model additionally included BMI, surgical history, employment status, place of residence, disease duration, and medication use. To assess the robustness of the disease-activity grouping decision, sensitivity analyses were conducted using the original four-category disease-activity variable, an ordinal coding of disease activity, and a model excluding disease activity. Regression results were reported as unstandardized coefficients (B), standard errors (SEs), standardized coefficients (β), 95% confidence intervals (CIs), and *P* values. Multicollinearity was assessed using variance inflation factors and tolerance values. Linearity, homoscedasticity, and normality of residuals were assessed using standard diagnostic procedures, and residual independence was evaluated using the Durbin–Watson statistic. All analyses were based on the 249 participants with complete data, and no statistical imputation was performed. All statistical tests were two-tailed, and *P* < 0.05 was considered statistically significant.

## 3 Results

### 3.1 Participant characteristics

A total of 258 patients with inflammatory bowel disease (IBD) were screened for eligibility. Five patients did not meet the eligibility criteria, leaving 253 eligible patients. Of these, two declined participation, and questionnaires were distributed to the remaining 251 consenting participants. Two participants did not complete and return a completed questionnaire: one left because of a scheduled clinical examination, and one withdrew from participation. Consequently, 249 complete questionnaires were returned. No questionnaire was excluded after return, and all 249 participants had complete data for the variables analyzed and were included in the final analysis. The mean age was 42.3 ± 14.5 years, and 50.2% were male. Crohn's disease and ulcerative colitis accounted for 51.0% and 49.0% of the sample, respectively. Most participants were married (61.8%), lived in urban areas (72.7%), and were employed (59.4%). Regarding disease activity, 4.4% of patients were in remission, 10.0% had mild disease activity, 58.2% had moderate disease activity, and 27.3% had severe disease activity. Detailed demographic and clinical characteristics are presented in Table 1.

**Table 1:** Demographic and clinical characteristics of patients with inflammatory bowel disease (*N* = 249).

Variable	Category/statistic	Value
Sex	Female	124 (49.8)
	Male	125 (50.2)
Age (years)	Mean ± SD	42.3 ± 14.5
Disease type	Crohn's disease	127 (51.0)
	Ulcerative colitis	122 (49.0)
Education level	Junior high school or below	28 (11.2)
	High school/technical secondary school	118 (47.4)
	College/undergraduate	83 (33.3)
	Master's degree or above	20 (8.0)
Marital status	Married	154 (61.8)
	Unmarried	65 (26.1)
	Divorced/widowed	30 (12.0)
Residence	Rural	68 (27.3)
	Urban	181 (72.7)
Living arrangement	Living alone	83 (33.3)
	Living with others	166 (66.7)
Employment status	Employed	148 (59.4)
	Student	47 (18.9)
	Unemployed	54 (21.7)
Monthly household income per capita (RMB/month)	< 3,000	7 (2.8)
	3,000–5,999	63 (25.3)
	6,000–8,999	126 (50.6)
	9,000–11,999	39 (15.7)
	≥12,000	14 (5.6)
Type of medical insurance	Rural cooperative medical insurance scheme	66 (26.5)
	Urban employee/resident medical insurance	165 (66.3)
	Self-pay	18 (7.2)
Disease duration (years)	3 months– < 1 year	18 (7.2)
	1– < 5	135 (54.2)
	5– < 10	79 (31.7)
	≥10	17 (6.8)
Cumulative number of IBD-related hospitalizations since diagnosis	Median (IQR)	3 (2–5)
Medication use	No	20 (8.0)
	Yes	229 (92.0)
Surgical history	No	177 (71.1)
	Yes	72 (28.9)
BMI (kg/m^2^)	< 18.5	131 (52.6)
	18.5–23.9	91 (36.5)
	≥24.0	27 (10.8)
Disease activity status	Remission	11 (4.4)
	Mild	25 (10.0)
	Moderate	145 (58.2)
	Severe	68 (27.3)
MFI-20 total score	Mean ± SD	53.8 ± 17.6

Data are presented as *n* (%) unless otherwise indicated. Age and the MFI-20 total score are presented as mean ± SD, and the cumulative number of IBD-related hospitalizations since diagnosis is presented as median (IQR). BMI was categorized as < 18.5, 18.5–23.9, and ≥24.0 kg/m^2^. Disease activity was assessed using the HBI for Crohn's disease and the SCCAI for ulcerative colitis, with disease-specific scores mapped to common disease-activity categories. BMI, body mass index; HBI, Harvey–Bradshaw Index; IBD, inflammatory bowel disease; IQR, interquartile range; MFI-20, Multidimensional Fatigue Inventory; SCCAI, Simple Clinical Colitis Activity Index; SD, standard deviation.

### 3.2 Univariate associations with TSK-17 total scores

Female patients reported significantly higher TSK scores than male patients (41.61 ± 10.15 vs. 36.10 ± 9.13, *t* = 4.509, *P* < 0.001). Age was not significantly correlated with TSK-17 total scores (r = 0.096, *P* = 0.129). Patients living in rural areas had higher TSK scores than those residing in urban areas (41.00 ± 9.89 vs. 38.03 ± 9.98, *t* = 2.095, *P* = 0.037). TSK-17 scores also differed across employment categories (F = 6.322, *P* = 0.002), with unemployed patients having the highest mean score (42.56 ± 10.64).

Regarding clinical characteristics, patients with a history of surgery had significantly higher TSK-17 scores than those without surgery (42.40 ± 10.95 vs. 37.40 ± 9.27, *t* = −3.663, *P* < 0.001). The cumulative number of IBD-related hospitalizations since diagnosis was positively correlated with TSK-17 total scores (Spearman's ρ = 0.326, *P* < 0.001).

TSK-17 total scores differed across disease activity categories (Welch's *F* = 7.123, *P* < 0.001), with the remission group having the lowest mean score (31.36 ± 11.58).

Significant differences in TSK scores were also found across BMI categories (*F* = 4.562, *P* = 0.011), and medical insurance types (*F* = 4.422, *P* = 0.013). Nonparametric sensitivity analyses were consistent with the corresponding parametric results. Other sociodemographic and clinical variables were not statistically significant. Detailed results are presented in Table 2.

**Table 2:** Univariate associations of sociodemographic and clinical variables with TSK-17 total scores (*N* = 249).

Variable	Category/statistic	*n* (%) or value	TSK-17 total score, mean ±SD	Test statistic	*P* value
Sex	Female	124 (49.8)	41.61 ± 10.15	*t* = 4.509	<0.001
	Male	125 (50.2)	36.10 ± 9.13		
Age (years)	Continuous, mean ± SD	42.3 ± 14.5	—	*r* = 0.096	0.129
Disease type	Crohn's disease	127 (51.0)	38.70 ± 9.17	Welch's *t* = −0.228	0.820
	Ulcerative colitis	122 (49.0)	38.99 ± 10.87		
Education level	Junior high school or below	28 (11.2)	41.39 ± 10.25	*F* = 1.495	0.216
	High school/technical secondary school	118 (47.4)	38.24 ± 9.34		
	College/undergraduate	83 (33.3)	39.57 ± 10.95		
	Master's degree or above	20 (8.0)	35.85 ± 9.10		
Marital status	Married	154 (61.8)	38.50 ± 10.05	*F* = 1.296	0.275
	Unmarried	65 (26.1)	38.38 ± 9.63		
	Divorced/widowed	30 (12.0)	41.60 ± 10.59		
Residence	Rural	68 (27.3)	41.00 ± 9.89	*t* = 2.095	0.037
	Urban	181 (72.7)	38.03 ± 9.98		
Living arrangement	Living alone	83 (33.3)	39.06 ± 9.83	*t* = 0.241	0.810
	Living with others	166 (66.7)	38.73 ± 10.14		
Employment status	Employed	148 (59.4)	37.16 ± 9.58	*F* = 6.322	0.002
	Student	47 (18.9)	39.89 ± 9.55		
	Unemployed	54 (21.7)	42.56 ± 10.64		
Monthly household income per capita (RMB/month)	<3,000	7 (2.8)	36.57 ± 9.38	*F* = 0.134	0.970
	3,000–5,999	63 (25.3)	38.89 ± 10.64		
	6,000–8,999	126 (50.6)	39.11 ± 10.15		
	9,000–11,999	39 (15.7)	38.54 ± 9.92		
	≥12,000	14 (5.6)	38.21 ± 7.35		
Type of medical insurance	Rural cooperative medical insurance scheme	66 (26.5)	41.89 ± 8.70	*F* = 4.422	0.013
	Urban employee/resident medical insurance	165 (66.3)	37.61 ± 10.16		
	Self-pay	18 (7.2)	38.94 ± 11.39		
Disease duration (years)	3 months– <1 year	18 (7.2)	36.44 ± 10.86	*F* = 1.850	0.139
	1– <5	135 (54.2)	38.50 ± 9.79		
	5– <10	79 (31.7)	38.90 ± 10.19		
	≥10	17 (6.8)	43.88 ± 9.45		
Surgical history	No	177 (71.1)	37.40 ± 9.27	*t* = −3.663	<0.001
	Yes	72 (28.9)	42.40 ± 10.95		
Cumulative number of IBD-related hospitalizations since diagnosis	Continuous, median (IQR)	3 (2–5)	—	*ρ = 0.326*	<0.001
Medication use	No	20 (8.0)	37.15 ± 12.81	Welch's *t* = −0.627	0.537
	Yes	229 (92.0)	38.99 ± 9.76		
Disease activity	Remission	11 (4.4)	31.36 ± 11.58	Welch's *F* = 7.123	<0.001
	Mild	25 (10.0)	32.44 ± 8.37		
	Moderate	145 (58.2)	39.88 ± 8.91		
	Severe	68 (27.3)	40.19 ± 11.28		
BMI (kg/m^2^)	<18.5	131 (52.6)	40.53 ± 10.07	*F* = 4.562	0.011
	18.5–23.9	91 (36.5)	37.49 ± 9.54		
	≥24.0	27 (10.8)	35.22 ± 10.10		

Data are presented as *n* (%), mean ± SD, or median (IQR), as appropriate. Pearson's correlation coefficient was used for age, whereas Spearman's rank correlation coefficient was used for the cumulative number of IBD-related hospitalizations since diagnosis. Welch's *t* test was used for disease type and medication use, and Welch's ANOVA was used for disease activity because of unequal variances. Other categorical comparisons were performed using independent-samples *t* tests or one-way ANOVA, as appropriate. For categorical comparisons, nonparametric sensitivity analyses using the Mann–Whitney U test or Kruskal–Wallis test yielded the same pattern of statistical significance. BMI, body mass index; IBD, inflammatory bowel disease; IQR, interquartile range; SD, standard deviation; TSK-17, 17-item Tampa Scale for Kinesiophobia.

### 3.3 Descriptive statistics of functional independence, fatigue, and kinesiophobia

Using the 37-point threshold for descriptive classification, 142 participants (57.0%) had scores at or above this level. The mean Barthel Index score was 71.23 ± 19.79, with observed scores ranging from 15 to 100. The mean Multidimensional Fatigue Inventory (MFI-20) score was 53.79 ± 17.64, with scores ranging from 26 to 93. The mean TSK-17 total score was 38.84 ± 10.02 (range: 22–64) (Table 3).

**Table 3:** Descriptive statistics of the main study measures (*N* = 249).

Measure	Number of items	Possible score range	Observed range	Mean ±SD
Barthel index (BI) total score	10	0–100	15–100	71.23 ± 19.79
Multidimensional fatigue inventory (MFI-20) total score	20	20–100	26–93	53.79 ± 17.64
Tampa scale for kinesiophobia (TSK-17) total score	17	17–68	22–64	38.84 ± 10.02

Higher BI scores indicate greater independence in basic activities of daily living. Higher MFI-20 and TSK-17 scores indicate greater fatigue and kinesiophobia, respectively. BI, Barthel Index; MFI-20, Multidimensional Fatigue Inventory; SD, standard deviation; TSK-17, 17-item Tampa Scale for Kinesiophobia.

### 3.4 Correlations among BI, MFI-20, and TSK-17 total scores

Pearson correlation analysis showed that the BI total score was negatively correlated with the TSK-17 total score (r = −0.647, *P* < 0.001). The MFI-20 total score was positively correlated with the TSK-17 total score (r = 0.245, *P* < 0.001). The BI and MFI-20 total scores were weakly negatively correlated (r = −0.133, *P* = 0.036) (Table 4).

**Table 4:** Pearson correlations among BI, MFI-20, and TSK-17 total scores (*N* = 249).

Measure	BI total score	MFI-20 total score	TSK-17 total score
BI total score	1		
MFI-20 total score	−0.133^*^	1	
TSK-17 total score	−0.647^**^	0.245^**^	1

Data are presented as Pearson's correlation coefficients. ^*^P < 0.05; ^**^P < 0.01 (two-tailed). BI, Barthel Index; MFI-20, Multidimensional Fatigue Inventory; TSK-17, 17-item Tampa Scale for Kinesiophobia.

### 3.5 Multivariable linear regression analyses of variables associated with TSK-17 total scores

The primary model was statistically significant [F_(10, 238)_ = 26.452, *P* < 0.001], accounting for 52.6% of the variance in TSK-17 total scores (R^2^ = 0.526; adjusted R^2^ = 0.506). Variance inflation factors ranged from 1.119 to 3.139, and the Durbin–Watson statistic was 1.699.

The BI total score had the largest standardized coefficient in absolute magnitude and was negatively associated with TSK-17 total scores (B = −0.284, β = – 0.561, 95% CI: −0.336 to −0.233; *P* < 0.001). Higher MFI-20 scores were associated with higher TSK-17 total scores (B = 0.097, β = 0.171, 95% CI: 0.041 to 0.154; *P* = 0.001). Female sex was associated with higher TSK-17 total scores (B = 3.130, β = 0.156, 95% CI: 1.270 to 4.990; *P* = 0.001). Moderate disease activity, compared with lower clinical activity, was associated with higher TSK-17 total scores (B = 3.554, β = 0.175, 95% CI: 0.781 to 6.327; *P* = 0.012). Compared with patients covered by the rural cooperative medical insurance scheme, those with urban employee or resident medical insurance had lower TSK-17 total scores (B = −3.000, β = −0.142, 95% CI: −5.087 to −0.913; *P* = 0.005). Age, cumulative number of IBD-related hospitalizations since diagnosis, disease type, the self-pay category, and severe disease activity were not statistically significant (all *P* > 0.05) (Table 5).

**Table 5:** Multiple linear regression analysis of variables associated with TSK-17 total scores (*N* = 249).

Variable	*B*	SE	β	95% CI for *B*	*t*	*P* value	VIF	Tolerance
Constant	49.595	3.267	—	43.159 to 56.031	15.181	< 0.001	—	—
Female sex (vs. male)	3.130	0.944	0.156	1.270 to 4.990	3.315	0.001	1.119	0.893
Age (years)	0.022	0.034	0.031	−0.045 to 0.088	0.642	0.521	1.188	0.842
Cumulative number of IBD-related hospitalizations since diagnosis	0.275	0.166	0.087	−0.052 to 0.602	1.655	0.099	1.403	0.713
Ulcerative colitis (vs. Crohn's disease)	1.935	1.084	0.097	−0.201 to 4.070	1.785	0.076	1.475	0.678
Urban employee/resident medical insurance (vs. rural cooperative medical insurance scheme)	−3.000	1.060	−0.142	−5.087 to −0.913	−2.831	0.005	1.261	0.793
Self-pay (vs. rural cooperative medical insurance scheme)	−2.511	1.915	−0.065	−6.284 to 1.261	−1.311	0.191	1.235	0.809
Moderate disease activity (vs. lower clinical activity)	3.554	1.408	0.175	0.781 to 6.327	2.525	0.012	2.421	0.413
Severe disease activity (vs. lower clinical activity)	−0.558	1.774	−0.025	−4.053 to 2.937	−0.315	0.753	3.139	0.319
BI total score	−0.284	0.026	−0.561	−0.336 to −0.233	−10.930	< 0.001	1.326	0.754
MFI-20 total score	0.097	0.029	0.171	0.041 to 0.154	3.404	0.001	1.272	0.786

The dependent variable was the TSK-17 total score. Lower clinical activity comprised remission and mild disease activity. Age and the cumulative number of IBD-related hospitalizations since diagnosis were entered as continuous variables. Categorical variables were dummy-coded, with male sex, Crohn's disease, the rural cooperative medical insurance scheme, and lower clinical activity as the respective reference categories. All variables were entered simultaneously. *R*^2^ = 0.526; adjusted *R*^2^ = 0.506; *F*_(10, 238)_ = 26.452, *P* < 0.001; Durbin–Watson statistic = 1.699. *B*, unstandardized regression coefficient; BI, Barthel Index; CI, confidence interval; IBD, inflammatory bowel disease; MFI-20, 20-item Multidimensional Fatigue Inventory; SE, standard error; TSK-17, 17-item Tampa Scale for Kinesiophobia; VIF, variance inflation factor; β, standardized regression coefficient.

The expanded covariate-adjusted model was also statistically significant (F_(20, 228)_ = 12.919, *P* < 0.001; R^2^ = 0.531; adjusted R^2^ = 0.490). After additional adjustment for BMI, surgical history, employment status, place of residence, disease duration, and medication use, the associations of BI and MFI-20 total scores with TSK-17 total scores remained statistically significant (B = −0.269, β = −0.531, *P* < 0.001; and B = 0.101, β = 0.178, *P* = 0.001, respectively). None of the additional covariates was statistically significant. Sensitivity analyses using alternative disease-activity coding approaches showed consistent directions and statistical significance for BI and MFI-20 total scores, whereas estimates for disease activity varied across model specifications (Supplementary Tables S1, S2).

## 4 Discussion

In this cross-sectional study of 249 patients with inflammatory bowel disease (IBD), lower independence in basic activities of daily living and greater fatigue were the most consistent correlates of kinesiophobia, with independence in basic activities of daily living showing the largest standardized coefficient in absolute magnitude. These associations remained statistically significant after broader adjustment for demographic and clinical characteristics and across alternative specifications of disease activity, whereas the association with disease activity varied across analytical approaches. Together, these findings highlight everyday functioning and symptom burden as important dimensions of kinesiophobia in IBD.

Patients with IBD often demonstrate reduced levels of physical activity despite evidence that appropriately prescribed exercise is generally safe and may provide physical and psychological benefits, particularly in quiescent or mildly active disease (34, 35). However, the effects of exercise on fatigue and other patient-reported outcomes remain heterogeneous, and avoidance of physical activity is still common in this population (35). Compared with musculoskeletal conditions where kinesiophobia is commonly linked to pain and fear of reinjury, movement-related fear in IBD may occur in the context of unpredictable gastrointestinal symptoms, persistent fatigue, reduced functional capacity, and concerns about symptom exacerbation (9, 36).

The association between independence in basic activities of daily living and kinesiophobia was particularly notable. The Barthel Index assesses the extent to which individuals can independently perform basic activities such as mobility, transfers, stair climbing, feeding, dressing, and toileting (21). One possible interpretation is that limitations in routine mobility or self-care may be accompanied by lower perceived physical capability and greater concern about fatigue, symptom worsening, or inability to complete an activity safely. This finding highlights the relevance of everyday functional independence to movement-related fear in patients with IBD.

Fatigue was another consistent correlate of kinesiophobia, in line with recent findings in patients with IBD (12). Fatigue is one of the most prevalent and disabling symptoms in IBD and may persist even when disease activity is clinically controlled (37, 38). Persistent fatigue may be accompanied by lower perceived capacity for physical activity and greater concern that activity will aggravate symptoms or interfere with essential daily tasks. Previous research has described fatigue, reduced physical activity, and physical deconditioning as closely interrelated experiences (19, 37).

The association between disease activity and kinesiophobia was less consistent. Although moderate disease activity was associated with greater kinesiophobia in the categorical models, severe disease activity was not, and no association was observed when disease activity was modeled ordinally. This pattern suggests that the association between disease activity and kinesiophobia may not follow a clear stepwise gradient. Clinical activity categories may not fully capture patients' fatigue, which may persist even during clinical remission (39). By contrast, independence in basic activities of daily living and fatigue remained consistently associated with kinesiophobia across the different analytical approaches.

Female sex and medical insurance type were additional correlates of kinesiophobia. Female participants reported greater kinesiophobia after adjustment for other variables. Previous studies have reported higher levels of anxiety and depression among women with IBD, as well as broader sex-related differences in pain perception, which may be relevant to perceived physical vulnerability and activity-related concerns (40, 41). Participants covered by urban employee or resident medical insurance reported lower levels of kinesiophobia than those covered by the rural cooperative medical insurance scheme. This finding identifies medical insurance type as a contextual correlate of kinesiophobia in this sample and warrants further investigation. However, the mechanisms underlying this association were not assessed in the present study.

Taken together, these findings support consideration of independence in basic activities of daily living and fatigue, alongside disease activity, when assessing kinesiophobia in IBD. Although control of inflammation remains central to IBD management, clinical disease activity alone may not fully capture patients' perceived ability or willingness to engage in physical activity. Patients with marked fatigue or reduced independence in basic activities of daily living may therefore warrant closer attention when physical activity is discussed, and recommendations may need to be tailored to their functional capacity, symptom burden, and movement-related concerns.

Several limitations should be considered. First, the cross-sectional design precludes causal inference and determination of the temporal direction of the observed associations. Second, recruitment from the inpatient ward of a single tertiary hospital, where most participants had moderate or severe disease activity, may limit generalizability to other IBD populations and healthcare settings. Third, reliance on patient-reported measures may have introduced reporting or common-method bias. Although the BI demonstrated good internal consistency in this sample, its measurement properties have not been specifically established in IBD populations, and it may not capture broader dimensions of physical functioning. Fourth, potentially relevant psychological and behavioral factors were not comprehensively assessed, and no objective measures of physical activity or exercise capacity were included, which may have contributed to residual confounding and limited interpretation of actual activity behavior. Finally, the small remission subgroup and the harmonization of HBI and SCCAI scores into common activity categories may have reduced the precision of disease-activity estimates. Because the TSK-17 threshold of 37 has not been specifically validated in IBD, it was used only for descriptive classification, whereas the regression analyses used continuous TSK-17 scores. Future multicenter longitudinal studies incorporating psychological and behavioral factors as well as objective measures of physical activity are warranted to further clarify these associations.

## 5 Conclusion

Kinesiophobia represents an important clinical concern in patients with IBD and should not be considered solely in relation to clinical disease activity. The consistent associations with lower independence in basic activities of daily living and greater fatigue underscore the importance of considering patients' everyday functioning and symptom burden when evaluating movement-related concerns. Incorporating these dimensions alongside disease activity may support a more comprehensive assessment and an individualized approach to physical activity in IBD care. These findings represent cross-sectional associations and do not establish causal or temporal relationships.

## Data Availability

The raw data supporting the conclusions of this article will be made available by the authors, without undue reservation.
