# Supplementary material for: Factors associated with kinesiophobia in patients with inflammatory bowel disease: associations with functional independence and fatigue
Source: Front Med (Lausanne). 2026 Aug 14;13:1900689. doi: 10.3389/fmed.2026.1900689 (PMC13521776; doi:10.3389/fmed.2026.1900689)
Supplement: Supplementary file 1 [file Table_1.docx]

Supplementary Table S1. Expanded covariate-adjusted linear regression model for TSK-17 total scores (N = 249)

| **Variable** | | ***B*** | **SE** | ***β*** | **95% CI for *B*** | ***t*** | ***P* value** | **VIF** | **Tolerance** |
| --- | --- | --- | --- | --- | --- | --- | --- | --- | --- |
|  | Constant | 47.284 | 3.814 | — | 39.769 to 54.799 | 12.398 | <0.001 | — | — |
|  | Female sex (vs male) | 3.116 | 0.975 | 0.156 | 1.195 to 5.037 | 3.196 | 0.002 | 1.155 | 0.866 |
|  | Age (years) | 0.021 | 0.039 | 0.030 | −0.056 to 0.098 | 0.533 | 0.594 | 1.517 | 0.659 |
|  | Cumulative number of IBD-related hospitalizations since diagnosis | 0.260 | 0.182 | 0.083 | −0.099 to 0.619 | 1.428 | 0.155 | 1.637 | 0.611 |
|  | Ulcerative colitis (vs Crohn’s disease) | 2.205 | 1.148 | 0.110 | −0.057 to 4.467 | 1.922 | 0.056 | 1.600 | 0.625 |
|  | Moderate disease activity (vs lower clinical activity) | 3.736 | 1.470 | 0.184 | 0.839 to 6.633 | 2.541 | 0.012 | 2.556 | 0.391 |
|  | Severe disease activity (vs lower clinical activity) | −0.691 | 1.856 | −0.031 | −4.348 to 2.966 | −0.372 | 0.710 | 3.326 | 0.301 |
|  | Urban employee/resident medical insurance (vs rural cooperative medical insurance scheme) | −2.935 | 1.199 | −0.139 | −5.298 to −0.572 | −2.448 | 0.015 | 1.563 | 0.640 |
|  | Self-pay (vs rural cooperative medical insurance scheme) | −2.302 | 1.978 | −0.060 | −6.199 to 1.595 | −1.163 | 0.246 | 1.276 | 0.784 |
|  | BI total score | −0.269 | 0.029 | −0.531 | −0.326 to −0.212 | −9.297 | <0.001 | 1.586 | 0.631 |
|  | MFI-20 total score | 0.101 | 0.030 | 0.178 | 0.042 to 0.160 | 3.381 | 0.001 | 1.353 | 0.739 |
|  | BMI <18.5 kg/m² (vs 18.5–23.9 kg/m²) | 0.615 | 1.056 | 0.031 | −1.466 to 2.696 | 0.582 | 0.561 | 1.352 | 0.739 |
|  | BMI ≥24.0 kg/m² (vs 18.5–23.9 kg/m²) | −0.049 | 1.660 | −0.002 | −3.320 to 3.222 | −0.030 | 0.976 | 1.295 | 0.772 |
|  | Surgical history: yes (vs no) | 0.921 | 1.155 | 0.042 | −1.355 to 3.197 | 0.798 | 0.426 | 1.333 | 0.750 |
|  | Student (vs employed) | 0.844 | 1.295 | 0.033 | −1.708 to 3.396 | 0.652 | 0.515 | 1.248 | 0.801 |
|  | Unemployed (vs employed) | 0.381 | 1.372 | 0.016 | −2.322 to 3.084 | 0.278 | 0.781 | 1.554 | 0.643 |
|  | Urban residence (vs rural residence) | 0.002 | 1.145 | 0.000 | −2.254 to 2.258 | 0.001 | 0.999 | 1.266 | 0.790 |
|  | Disease duration 3 months–<1 year (vs 1–<5 years) | −0.337 | 2.026 | −0.009 | −4.329 to 3.655 | −0.166 | 0.868 | 1.339 | 0.747 |
|  | Disease duration 5–<10 years (vs 1–<5 years) | −0.287 | 1.063 | −0.013 | −2.382 to 1.808 | −0.270 | 0.788 | 1.189 | 0.841 |
|  | Disease duration ≥10 years (vs 1–<5 years) | 1.357 | 2.111 | 0.034 | −2.803 to 5.517 | 0.643 | 0.521 | 1.378 | 0.726 |
|  | No medication use (vs use) | 0.426 | 1.835 | 0.012 | −3.190 to 4.042 | 0.232 | 0.817 | 1.210 | 0.827 |

**Notes:** The dependent variable was the TSK-17 total score. The expanded model included all variables in the primary model and additionally adjusted for BMI, surgical history, employment status, place of residence, disease duration, and medication use. Lower clinical activity comprised remission and mild disease activity. Age and the cumulative number of IBD-related hospitalizations since diagnosis were entered as continuous variables. Categorical variables were dummy-coded, with male sex, Crohn’s disease, the rural cooperative medical insurance scheme, lower clinical activity, BMI of 18.5–23.9 kg/m², no surgical history, employed status, rural residence, disease duration of 1–<5 years, and medication use as the respective reference categories. All variables were entered simultaneously. *R*² = 0.531; adjusted *R*² = 0.490; *F*(20, 228) = 12.919, *P* < 0.001; Durbin–Watson statistic = 1.733. *B*, unstandardized regression coefficient; BI, Barthel Index; BMI, body mass index; CI, confidence interval; IBD, inflammatory bowel disease; MFI-20, 20-item Multidimensional Fatigue Inventory; SE, standard error; TSK-17, 17-item Tampa Scale for Kinesiophobia; VIF, variance inflation factor; *β*, standardized regression coefficient.
