# Supplementary material for: Factors associated with kinesiophobia in patients with inflammatory bowel disease: associations with functional independence and fatigue
Source: Front Med (Lausanne). 2026 Aug 14;13:1900689. doi: 10.3389/fmed.2026.1900689 (PMC13521776; doi:10.3389/fmed.2026.1900689)
Supplement: Supplementary file 2 [file Table_2.docx]

**Supplementary Table S2. Sensitivity analyses using alternative specifications of disease activity**

**Panel A. Original four-category disease-activity specification**

| **Variable** | ***B*** | **SE** | ***β*** | **95% CI for *B*** | ***t*** | ***P* value** | **VIF** | **Tolerance** |
| --- | --- | --- | --- | --- | --- | --- | --- | --- |
| Constant | 47.595 | 3.818 | — | 40.073 to 55.117 | 12.465 | <0.001 | — | — |
| Female sex (vs male) | 3.095 | 0.945 | 0.155 | 1.234 to 4.956 | 3.276 | 0.001 | 1.121 | 0.892 |
| Age (years) | 0.022 | 0.034 | 0.031 | −0.045 to 0.088 | 0.642 | 0.522 | 1.188 | 0.842 |
| Cumulative number of IBD-related hospitalizations since diagnosis | 0.284 | 0.166 | 0.090 | −0.044 to 0.611 | 1.706 | 0.089 | 1.407 | 0.711 |
| Ulcerative colitis (vs Crohn’s disease) | 2.262 | 1.131 | 0.113 | 0.033 to 4.491 | 2.000 | 0.047 | 1.607 | 0.622 |
| Mild disease activity (vs remission) | 2.709 | 2.677 | 0.081 | −2.564 to 7.981 | 1.012 | 0.313 | 3.251 | 0.308 |
| Moderate disease activity (vs remission) | 5.422 | 2.322 | 0.267 | 0.849 to 9.996 | 2.336 | 0.020 | 6.586 | 0.152 |
| Severe disease activity (vs remission) | 1.096 | 2.413 | 0.049 | −3.657 to 5.849 | 0.454 | 0.650 | 5.805 | 0.172 |
| Urban employee/resident medical insurance (vs rural cooperative medical insurance scheme) | −3.045 | 1.060 | −0.144 | −5.134 to −0.956 | −2.872 | 0.004 | 1.263 | 0.792 |
| Self-pay (vs rural cooperative medical insurance scheme) | −2.675 | 1.922 | −0.069 | −6.461 to 1.111 | −1.392 | 0.165 | 1.244 | 0.804 |
| BI total score | −0.285 | 0.026 | −0.562 | −0.336 to −0.233 | −10.941 | <0.001 | 1.326 | 0.754 |
| MFI-20 total score | 0.099 | 0.029 | 0.174 | 0.042 to 0.155 | 3.449 | 0.001 | 1.275 | 0.785 |

**Model statistics:** R² = 0.528; adjusted R² = 0.507; F(11, 237) = 24.143, P < 0.001; Durbin–Watson statistic = 1.695.

**Panel B. Ordinal coding of disease activity**

| **Variable** | ***B*** | **SE** | ***β*** | **95% CI for *B*** | ***t*** | ***P* value** | **VIF** | **Tolerance** |
| --- | --- | --- | --- | --- | --- | --- | --- | --- |
| Constant | 53.691 | 3.324 | — | 47.142 to 60.239 | 16.152 | <0.001 | — | — |
| Female sex (vs male) | 2.722 | 0.962 | 0.136 | 0.827 to 4.618 | 2.829 | 0.005 | 1.100 | 0.909 |
| Age (years) | 0.027 | 0.035 | 0.039 | −0.041 to 0.095 | 0.783 | 0.434 | 1.186 | 0.843 |
| Cumulative number of IBD-related hospitalizations since diagnosis | 0.215 | 0.170 | 0.068 | −0.119 to 0.550 | 1.266 | 0.207 | 1.388 | 0.721 |
| Ulcerative colitis (vs Crohn’s disease) | 0.360 | 1.006 | 0.018 | −1.622 to 2.342 | 0.358 | 0.721 | 1.202 | 0.832 |
| Disease activity (ordinal) | −0.039 | 0.736 | −0.003 | −1.488 to 1.411 | −0.053 | 0.958 | 1.397 | 0.716 |
| Urban employee/resident medical insurance (vs rural cooperative medical insurance scheme) | −3.320 | 1.082 | −0.157 | −5.452 to −1.188 | −3.067 | 0.002 | 1.244 | 0.804 |
| Self-pay (vs rural cooperative medical insurance scheme) | −2.921 | 1.954 | −0.076 | −6.771 to 0.928 | −1.495 | 0.136 | 1.217 | 0.822 |
| BI total score | −0.289 | 0.027 | −0.570 | −0.341 to −0.236 | −10.825 | <0.001 | 1.316 | 0.760 |
| MFI-20 total score | 0.087 | 0.029 | 0.152 | 0.030 to 0.144 | 2.988 | 0.003 | 1.238 | 0.808 |

**Model statistics:** N = 249; R² = 0.497; adjusted R² = 0.478; F(9, 239) = 26.259, P < 0.001; Durbin–Watson statistic = 1.711.

**Panel C. Model excluding disease activity**

| **Variable** | ***B*** | **SE** | ***β*** | **95% CI for *B*** | ***t*** | ***P* value** | **VIF** | **Tolerance** |
| --- | --- | --- | --- | --- | --- | --- | --- | --- |
| Constant | 53.620 | 3.039 | — | 47.633 to 59.607 | 17.643 | <0.001 | — | — |
| Female sex (vs male) | 2.727 | 0.956 | 0.136 | 0.844 to 4.610 | 2.853 | 0.005 | 1.089 | 0.918 |
| Age (years) | 0.027 | 0.034 | 0.039 | −0.041 to 0.095 | 0.790 | 0.431 | 1.181 | 0.847 |
| Cumulative number of IBD-related hospitalizations since diagnosis | 0.214 | 0.169 | 0.068 | −0.118 to 0.547 | 1.269 | 0.206 | 1.379 | 0.725 |
| Ulcerative colitis (vs Crohn’s disease) | 0.343 | 0.953 | 0.017 | −1.535 to 2.221 | 0.360 | 0.719 | 1.084 | 0.923 |
| Urban employee/resident medical insurance (vs rural cooperative medical insurance scheme) | −3.312 | 1.070 | −0.157 | −5.419 to −1.205 | −3.096 | 0.002 | 1.220 | 0.819 |
| Self-pay (vs rural cooperative medical insurance scheme) | −2.905 | 1.926 | −0.075 | −6.699 to 0.889 | −1.508 | 0.133 | 1.187 | 0.843 |
| BI total score | −0.288 | 0.026 | −0.569 | −0.340 to −0.237 | −11.025 | <0.001 | 1.272 | 0.786 |
| MFI-20 total score | 0.086 | 0.027 | 0.151 | 0.033 to 0.139 | 3.223 | 0.001 | 1.054 | 0.949 |

**Model statistics:** R² = 0.497; adjusted R² = 0.480; F(8, 240) = 29.665, P < 0.001; Durbin–Watson statistic = 1.711.

**Notes:** The dependent variable was the TSK-17 total score. All models included sex, age, the cumulative number of IBD-related hospitalizations since diagnosis, disease type, type of medical insurance, BI total score, and MFI-20 total score. Panel A entered disease activity as the original four-category variable, with remission as the reference category. The higher VIFs for moderate and severe disease activity in Panel A may reflect instability related to the small remission reference group (n = 11); the maximum VIF was reduced to 3.139 in the primary three-category model. Panel B entered disease activity as an ordinal variable coded as remission = 0, mild = 1, moderate = 2, and severe = 3. Panel C excluded disease activity. Age and the cumulative number of IBD-related hospitalizations since diagnosis were entered as continuous variables. Categorical variables were dummy-coded, with male sex, Crohn’s disease, and the rural cooperative medical insurance scheme as the respective reference categories. All variables specified for each model were entered simultaneously. B, unstandardized regression coefficient; BI, Barthel Index; CI, confidence interval; IBD, inflammatory bowel disease; MFI-20, 20-item Multidimensional Fatigue Inventory; SE, standard error; TSK-17, 17-item Tampa Scale for Kinesiophobia; VIF, variance inflation factor; β, standardized regression coefficient.
